# Supplementary material for: Role of Baseline Gut Microbiota on Response to Fiber Intervention in Individuals with Irritable Bowel Syndrome
Source: Nutrients. 2023 Nov 15;15(22):4786. doi: 10.3390/nu15224786 (PMC10674363; doi:10.3390/nu15224786)
Supplement: Supplementary file 1 [file nutrients-15-04786-s001.zip › nutrients-2687798-supplementary.pdf]

Supplementary Materials

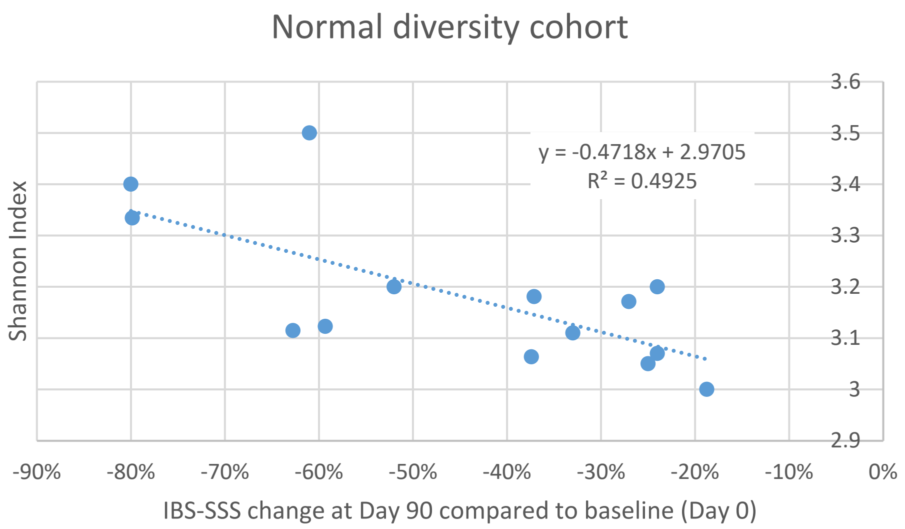

Figure S1. Correlation between microbiota diversity (Shannon Index) and percentage change to IBS symptom severity score in the normal baseline microbiota diversity cohort.

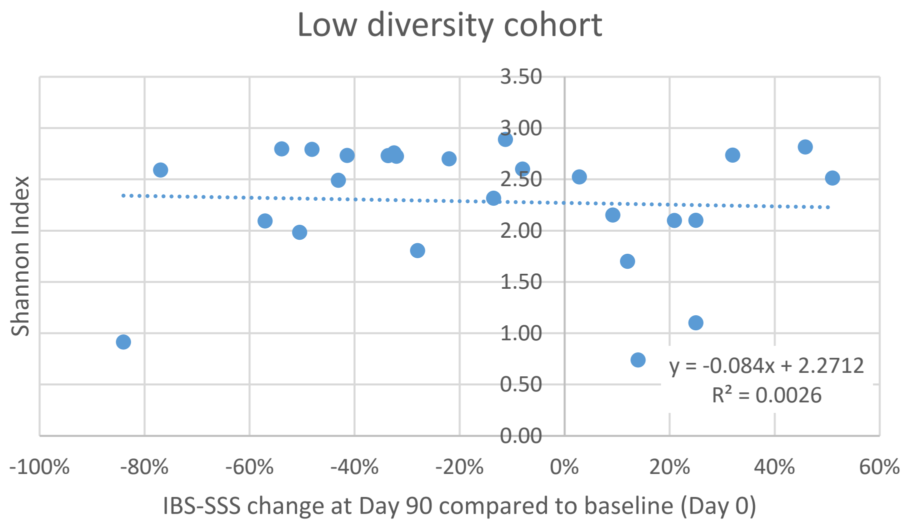

Figure S2. Correlation between microbiota diversity (Shannon Index) and percentage change to IBS symptom severity score in the low baseline microbiota diversity cohort.

Table S1. Differential microbes between group responsive to PHGG and not responsive to PHGG at Day 30 of intervention.

Phylum	Bacteria	Responsive to PHGG (% ±SD)	No Response to PHGG (% ±SD)	*p* Value *	FDR	LDA Score
Firmicutes	*Oscillospira*	5.86 ± 4.71	1.47 ± 1.02	<0.001	0.001	5.83
Bacteroidetes	*Odoribacter*	2.32 ± 2.76	0.11 ± 0.10	<0.001	0.004	5.12
Firmicutes	*F. prausnitzii*	7.23 ± 5.76	17.71 ± 14.83	0.0033	0.0351	−5.82
Bacteroidetes	*P. copri*	0.07 ± 0.12	14.02 ± 27.33	0.0035	0.0452	−5.64
Bacteroidetes	*Prevotella*	0.08 ± 0.20	15.83 ± 26.62	0.0035	0.0440	−5.93
